# D-tyrosine adds an anti-melanogenic effect to cosmetic peptides

**DOI:** 10.1038/s41598-019-57159-3

**Published:** 2020-01-14

**Authors:** Jisu Park, Hyejung Jung, Bohee Jang, Hyun-Kuk Song, Inn-Oc Han, Eok-Soo Oh

**Affiliations:** 1 Department of Life Sciences, the Research Center for Cellular Homeostasis, Ewha Womans University, Seoul, 03760 Korea; 2 Skin QC Institute of Dermatological Sciences, Seoul, Korea; 3 Department of Physiology and Biophysics, College of Medicine, Inha University, Incheon, 22212 Korea

**Keywords:** Biochemistry, Cell biology, Chemical biology

## Abstract

D-tyrosine is known to negatively regulate melanin synthesis by inhibiting tyrosinase activity. Here, we further reveal that peptides containing terminal D-tyrosine can reduce the melanin contents of human melanocytes. The addition of D-tyrosine to the terminus of the commercial anti-wrinkle peptide, pentapeptide-18 endowed the peptide with the ability to reduce the melanin content and tyrosinase activity in human MNT-1 melanoma cells and primary melanocytes. Consistently, terminal D-tyrosine-containing pentapeptide-18 inhibited the melanogenesis induced by α-MSH treatment or UV irradiation of MNT-1 cells and reduced melanin synthesis in the epidermal basal layer of a 3D human skin model. Furthermore, the addition of D-tyrosine to an anti-aging peptide (GEKG) or an anti-inflammatory peptide (GHK) endowed these short peptides with anti-melanogenic effects without altering their intrinsic effects. Together, these data suggest that the addition of D-tyrosine at the terminus of a short cosmetic peptide adds an anti-melanogenic effect to its intrinsic cosmetic effect. Our work offers a novel means of generating dual-function cosmetic peptides.

## Introduction

Melanin synthesis occurs in melanocytes and is an essential physiological process that determines the color of human skin and protects its DNA from UV damage^1^. It is closely related with the occurrence of pigmentary disorders^2^: the imbalanced regulation of melanin synthesis results in many pigmentary skin diseases that commonly affect men and women of all ethnic groups^3^, including hyperpigmentation disorders, such as melanocytic nevus, seborrheic keratosis, and melanoma, and hypopigmentation disorders, such as piebaldism, pityriasis, and vitiligo.

Melanin is synthesized in melanosomes, which are transferred to surrounding keratinocytes where they protect cells against DNA damage^4,5^. Melanogenesis is critically regulated by the expression of various melanogenesis-related enzymes, such as tyrosinase, tyrosinase-related protein 1 (TRP-1), and tyrosinase-related protein 2 (TRP-2). Tyrosinase, the rate-limiting enzyme for controlling melanin synthesis, is involved primarily in the production of L-dopaquinone^5,6^. TRP-1 and TRP-2 catalyze specific steps in melanogenesis and stabilize tyrosinase activity^7^. As tyrosinase is a key enzyme that catalyzes a rate-limiting step of melanin synthesis, numerous inhibitors that target tyrosinase have been investigated for their ability to inhibit this process. These include well-known tyrosinase inhibitors, such as hydroquinone^8^, arbutin^9^, kojic acid^10^, and salicylic acid^11^. However, due to the side effects of these inhibitors and the increasing demand for safe and effective cosmetics, many continuing efforts are being made to identify or produce new skin-whitening agents. Some researchers have screened natural products and found that chalcones, resveratrol, and coumarins exhibit inhibitory activity against mushroom tyrosinase^12^. Other groups have sought to develop bioactive materials. Particular attention has been paid to various bioactive peptides, including short-sequence oligopeptides^13^, kojic acid-tripeptide compounds^14^, dipeptides and tripeptides derived from natural products^15^, and cysteine-containing dipeptides^16^, all of which have been used as cosmetic peptides. In the context of topical application, peptides have strong advantages due to their stability, easy synthesis and modification, and diverse availability. For example, the tetrapeptide, PKEK, inhibits the UVB-induced upregulation of the genes encoding IL-1α, IL-6, IL-8, and TNF-α and the protein levels of POMC and tyrosinase, and it may be suitable as a skin tone-modulating agent in cosmetic products^17^. At present, bioactive peptides are widely used by cosmeceutical companies as cosmetics.

We recently reported that D-tyrosine, the enantiomer of L-tyrosine, suppresses melanogenesis induced by α-MSH treatment or UV irradiation, two key inducers of melanogenesis, in melanocytes by inhibiting the enzymatic activity of tyrosinase^18^. Based on this, we hypothesized that the presence of D-tyrosine could enable a cosmetic peptide to negatively regulate melanin synthesis. Here, we investigated whether D-tyrosine-containing cosmetic peptides can regulate melanin synthesis in melanoma cells and melanocytes.

## Results

### Terminal D-tyrosine endows an anti-wrinkle peptide with an additional anti-melanogenic effect

**Figure 1:**
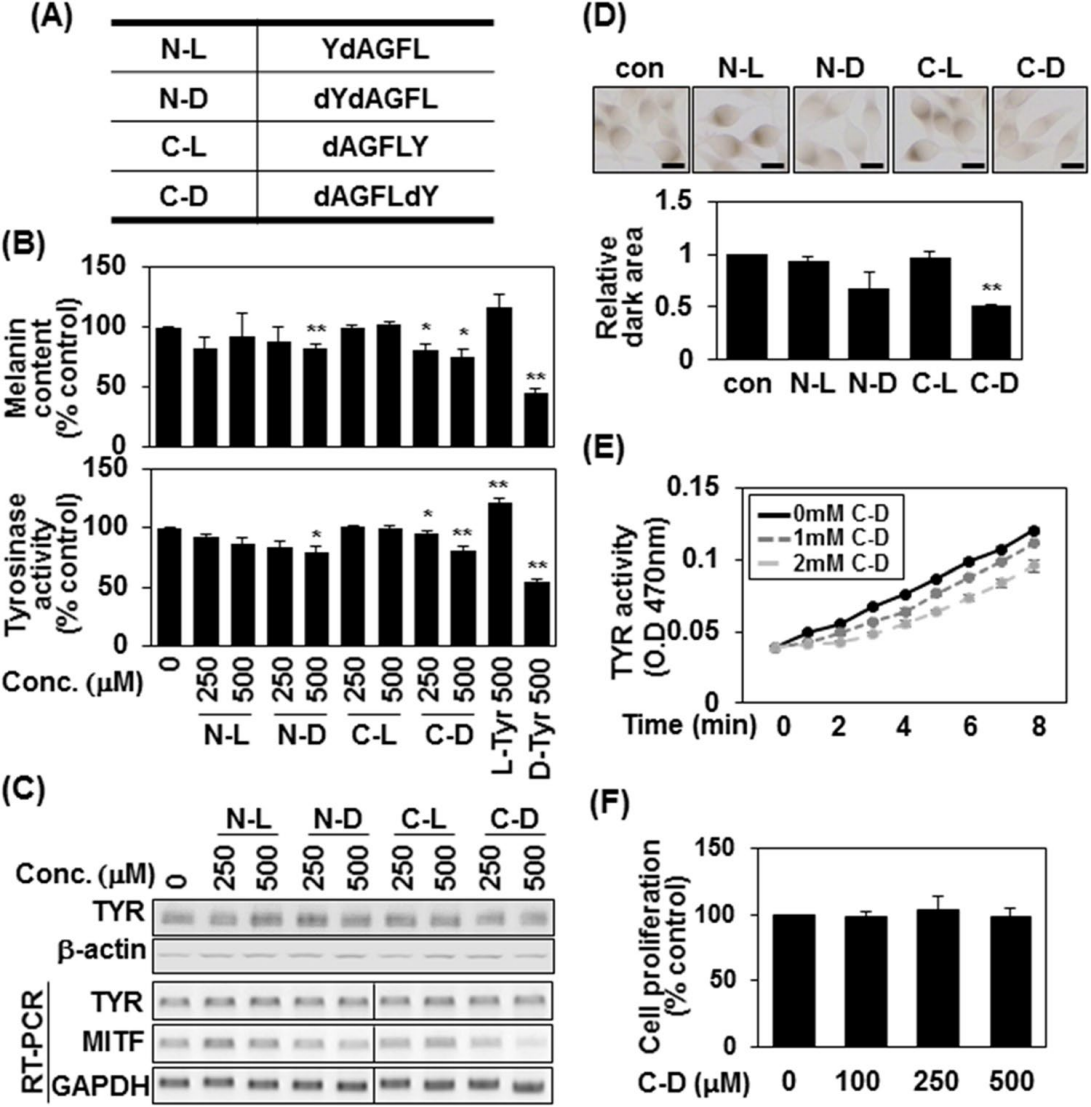
Pentapeptide-18 containing terminal D-tyrosine inhibits melanin synthesis. (**A**) Schematic diagram depicting the versions of pentapeptide-18 (YdAGFL) synthesized herein, including N-terminal L-Tyr (N-L) or D-Tyr (N-D) and/or C-terminal L-Tyr (C-L) or D-Tyr (C-D). (**B**) MNT-1 cells were treated with the indicated amount of peptide or L- or D-tyrosine for 24 h. The melanin content was measured by absorbance at 405 nm and is given as the mean of three independent experiments ± SD; *P < 0.05 and **P < 0.01 (top panel). Cell lysates (100 μg) were reacted with L-DOPA at 37 °C for 3 h and tyrosinase activity was determined at 470 nm. The mean percentages (n = 3) ± SD are shown; *P < 0.05 and **P < 0.01 (bottom panel). (**C**) Cells were lysed with RIPA buffer and protein expression was measured by Western blot analysis (top panel) or mRNA expression was analyzed by RT-PCR (bottom panel). Shown are cropped gels (Cropped gels indicated cropping lines are also shown in Supplementary Fig. S1). (**D**) MNT-1 cells were plated on 12-well plates, treated with 500 μM of the indicated peptides for 24 h, and reacted with L-DOPA at 37 °C for 3 h. Bright-field microscopic images are shown (top panel). Scale bars = 20 μm. Relative amounts of stained regions were measured with the ImageJ program (bottom panel). **P < 0.01. (**E**) Mushroom tyrosinase (5 unit) was incubated at 37 °C in buffer containing L-tyrosine (250 μM) with different concentrations of C-D peptide. Reaction rates were calculated as the absorbance change (470 nm)/min. (**F**) MNT-1 cells were incubated with the indicated concentrations of D- or L-tyrosine, and cell viability was determined by MTT assay. Data are shown as mean ± S.D. (n = 3), *p < 0.05, **p < 0.01 versus DW control or peptide.

Our observation that D-tyrosine negatively regulates melanin synthesis^18^ prompted us to further investigate whether a D-tyrosine-containing peptide could have a similar anti-melanogenic effect (Fig. 1). We selected Leuphasyl (pentapeptide-18; YdAGFL), a commercialized cosmetic peptide that acts as a neurotransmitter inhibitor to reduce fine lines and wrinkles^19,20^. We synthesized pentapeptide-18 peptides in which we either replaced L-tyrosine with D-tyrosine at the N-terminus or added a L- or D-tyrosine at the C-terminus (Fig. 1A) and treated human melanoma MNT-1 cells with various doses of each peptide (Fig. 1B). Expectedly, 500 μM of D-tyrosine reduced the melanin content of MNT-1 cells by about 50% (Fig. 1B). Notably, 500 μM of peptapeptide-18 containing D-tyrosine at the N-terminus (N-D) or C-terminus (C-D) decreased the melanin contents and tyrosinase activities of MNT-1 cells by 18% and 25%, respectively (Fig. 1B). Consistently, 500 μM of N-D or C-D decreased the expression of tyrosinase and microphthalmia-associated transcription factor (MITF), which plays a critical role in melanogenesis (Fig. 1C). L-DOPA staining also showed that the level of intracellular tyrosinase activity was decreased in MNT-1 cells treated with 500 μM of N-D or C-D (Fig. 1D), and tyrosinase activity was reduced by C-D treatment in *in vitro* (Fig. 1E). However, MTT assays showed that 500 μM of N-D or C-D did not affect the proliferation of MNT-1 cells (Fig. 1F). Together, these data suggest that pentapeptide-18 containing terminal D-tyrosine inhibits melanin synthesis in human melanoma cells.

### Pentapeptide-18 containing terminal D-tyrosine suppresses melanogenesis induced by α-MSH and UVB

**Figure 2:**
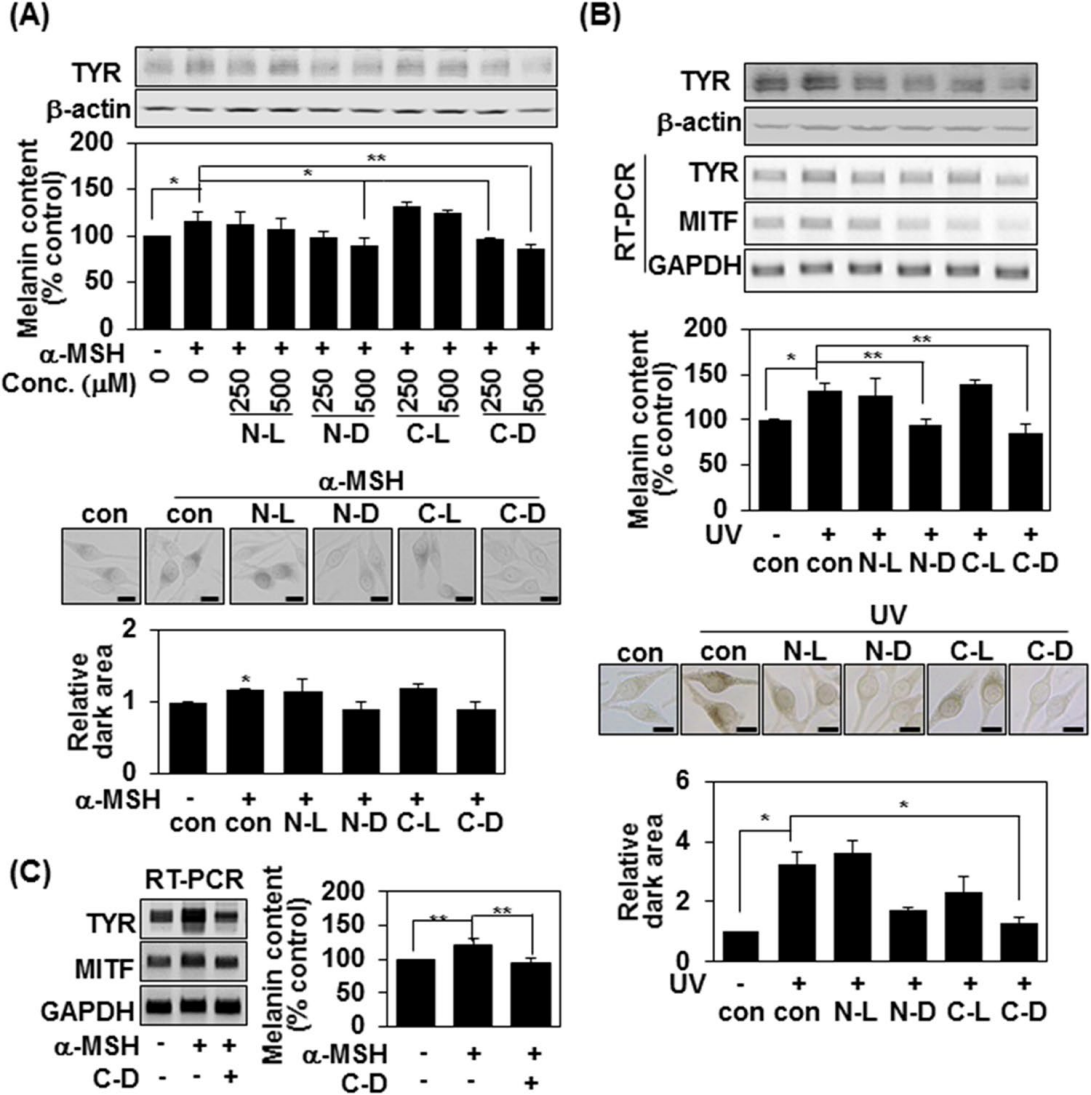
Pentapeptide-18 with terminal D-tyrosine suppresses the melanogenesis induced by α-MSH and UVB. (**A**,**B**) MNT-1 cells were either treated with 1 μM of α-MSH plus the indicated concentrations of peptides for 24 h (A) or irradiated with 100 mJ/cm^2^ UVB and treated with 500 μM of peptides for 24 h (**B**). Protein expression was measured by Western blot analysis and mRNA expression was analyzed by RT-PCR (top panel). The mean percentages of melanin content ± SD are shown; *P < 0.05 and **P < 0.01 (middle panel). MNT-1 cells were plated on 12-well plates, treated with 500 μM of indicated peptide plus α-MSH (1 μM) for 24 h, or irradiated with 100 mJ/cm^2^ UVB and treated with 500 μM of peptides for 24 h and reacted with L-DOPA at 37 °C for 3 h. Bright-field microscopic images are shown. Scale bars = 20 μm. Relative amounts of stained regions were measured with the ImageJ program; *P < 0.05 (bottom panel). (**C**) MNT-1 cells were either treated with 100 nM of α-MSH with/without 250 μM of C-D for 72 h. mRNA expression was analyzed by RT-PCR (left panel). The mean percentages of melanin content ± SD are shown; *P < 0.05 and **P < 0.01 (right panel).

As α-MSH is known to be a key regulator of melanogenesis in melanocytes^21^, we investigated whether the D-tyrosine-containing peptides could suppress α-MSH-induced melanin synthesis. Indeed, 500 μM of N-D and C-D suppressed the α-MSH-induced increases of tyrosinase in MNT-1 cells (Fig. 2A, top). Similarly, N-D and C-D downregulated the α-MSH-induced increases of melanin synthesis and intracellular tyrosinase activity, as assessed by L-DOPA staining (Fig. 2A, bottom). Since ultraviolet (UV) irradiation is also known to stimulate melanogenesis in the skin, we examined whether N-D and C-D could suppress UV-induced melanin synthesis. 500 μM of N-D and C-D suppressed the UV-induced increases in tyrosinase protein expression and the mRNA levels of tyrosinase and MITF (Fig. 2B, top). Similarly, N-D and C-D down-regulated the UV-induced increases in melanin synthesis and intracellular tyrosinase activity (Fig. 2B, bottom). In addition, 250 μM of C-D suppressed the α-MSH (100 nM)-induced increases of tyrosinase and MITF mRNA levels and melanin synthesis in MNT-1 cells (Fig. 2C), confirming that terminal D-Tyrosine-containing pentapeptide-18 can regulate the melanogenesis triggered by α-MSH and UV irradiation, which are the most common environmental factors that cause melanin synthesis.

### Terminal D-tyrosine-containing pentapeptide-18 inhibits melanogenesis in human melanocytes

**Figure 3:**
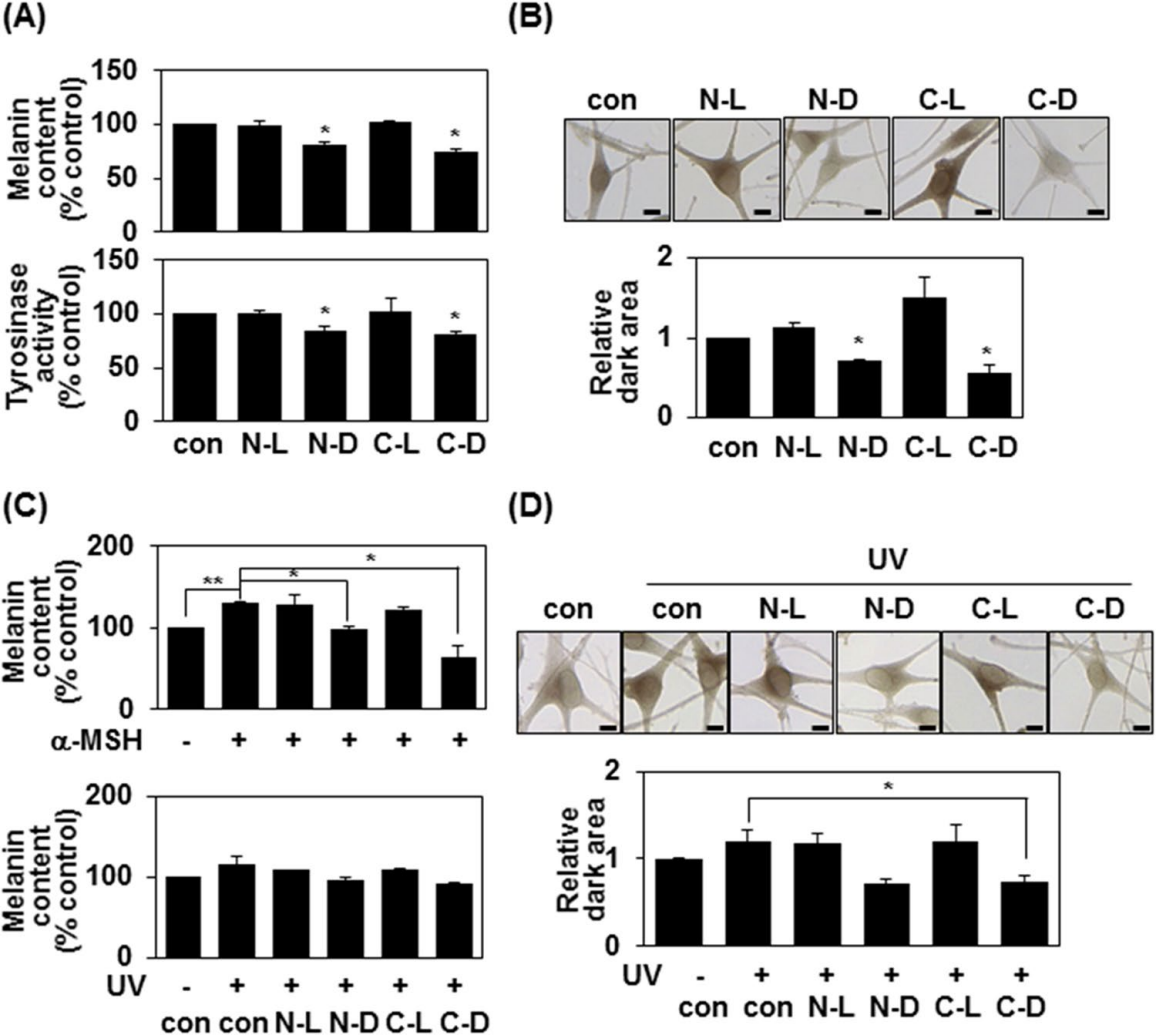
Pentapeptide-18 with terminal D-tyrosine inhibits melanin synthesis in human melanocytes. (**A**) Human melanocytes were incubated with 500 μM of the indicated peptides for 24 h. The melanin content was measured by absorbance at 405 nm and is given as the mean of three independent experiments ± SD; *P < 0.05 (top panel). Cell lysates (80 μg) were reacted with L-DOPA at 37 °C for 30 min, and tyrosinase activity was determined at 470 nm. The mean percentages (n = 3) ± SD are shown; *P < 0.05 (bottom panel). (**B**) Human melanocytes plated to 12-well plates were treated with 500 μM of the indicated peptides for 24 h and reacted with L-DOPA at 37 °C for 30 min. Bright-field microscopic images are shown (top panel). Scale bars = 20 μm. Relative amounts of stained regions were measured using the ImageJ program (bottom panel). *P < 0.05. (**C**) Human melanocytes were either treated with α-MSH (1 μM) plus 500 μM of the indicated peptide for 24 h (top panel), or irradiated with 100 mJ/cm^2^ UVB and treated with 500 μM of peptide for 24 h (bottom panel). Melanin contents were measured by absorbance at 405 nm. The mean percentages (n = 3) ± SD are shown; *P < 0.05 and **P < 0.01. (**D**) Human melanocytes distributed to 12-well plates were irradiated with 100 mJ/cm^2^ UVB, treated with 500 μM of peptide for 24 h, and reacted with L-DOPA at 37 °C for 30 min. Bright-field microscopic images are shown (top panel). Scale bars = 20 μm. Relative amounts of stained regions were measured using the ImageJ program (bottom panel). *P < 0.05.

**Figure 4:**
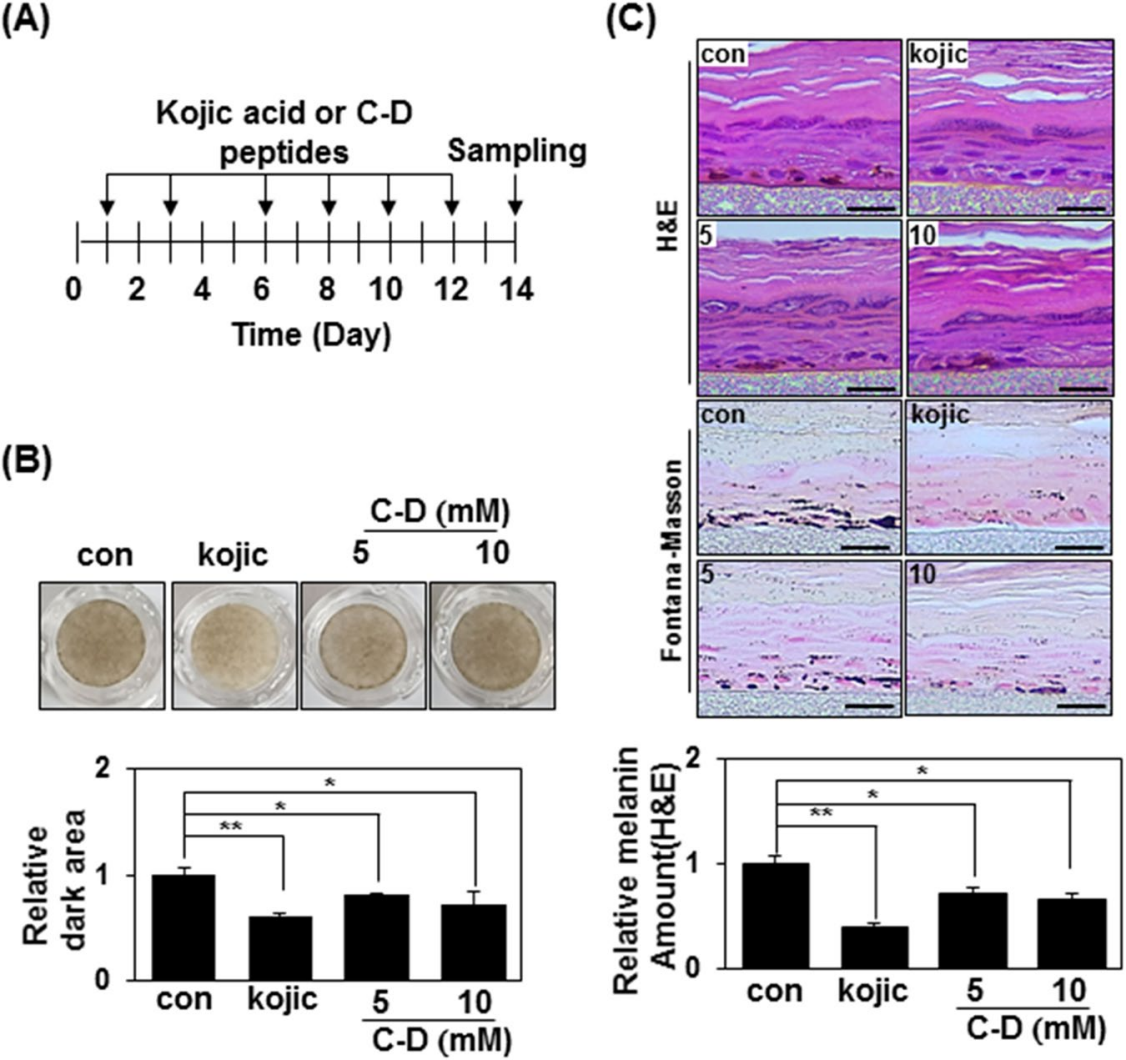
Terminal D-tyrosine-containing pentapeptide-18 decreases melanin synthesis in human skin. (**A**) Schematic of the experimental strategy. Melanoderm skin models were treated with distilled PBS (control), 5 or 10 mM C-D peptide, or 1% of Kojic acid (50 μl each) eight times over 14 days. On day 14, the samples were embedded in paraffin and sectioned at 4 μm. (**B**) Macroscopic views of the cultured human skin model (top panel). Skin darkness value was analyzed using the ImageJ program (bottom panel). *P < 0.05. (**C**) Paraffin-embedded tissue sections were either stained using hematoxylin and eosin (top panel) or Fontana-Masson (middle panel). Scale bars = 50 μm. Relative amounts of melanin in hematoxylin and eosin-stained sections were measured with the ImageJ program (bottom panel). **P < 0.01.

Consistent with the results obtained in human melanoma cells, treatment of human melanocytes with 500 μM of N-D or C-D triggered decreases in melanin synthesis, total tyrosinase activity (Fig. 3A), and intracellular tyrosinase activity (Fig. 3B). N-D and C-D also inhibited α-MSH and UV-mediated melanin synthesis in this system (Fig. 3C,D), indicating that terminal D-tyrosine-containing pentapeptide-18 decreases melanin synthesis in human primary melanocytes. We further investigated anti-melanogenic activity of the C-D peptide using a 3D model of human epidermis (Fig. 4). MelanoDerm tissues models were treated with 50 μl of distilled PBS (control), the indicated amount of C-D peptide, or 1% of kojic acid as a positive control, every other day for 14 days (Fig. 4A). At the end of this period, MelanoDerm tissues samples were embedded in paraffin and sectioned at 4 μm. In macroscopic images, reduced pigmentation was seen in the C-D peptides-treated skin samples compared with PBS-treated samples (Fig. 4B). Both H&E staining and Fontana-Masson staining revealed that the amounts of melanin were significantly lower in D-tyrosine-containing peptides treated skin samples (Fig. 4C). Taken together, these results suggest that addition of D-tyrosine may add additional anti-melanogenic effect to the cosmetic peptides.

### D-tyrosine gives short commercial cosmetic peptides an additional anti-melanogenic effect

**Figure 5:**
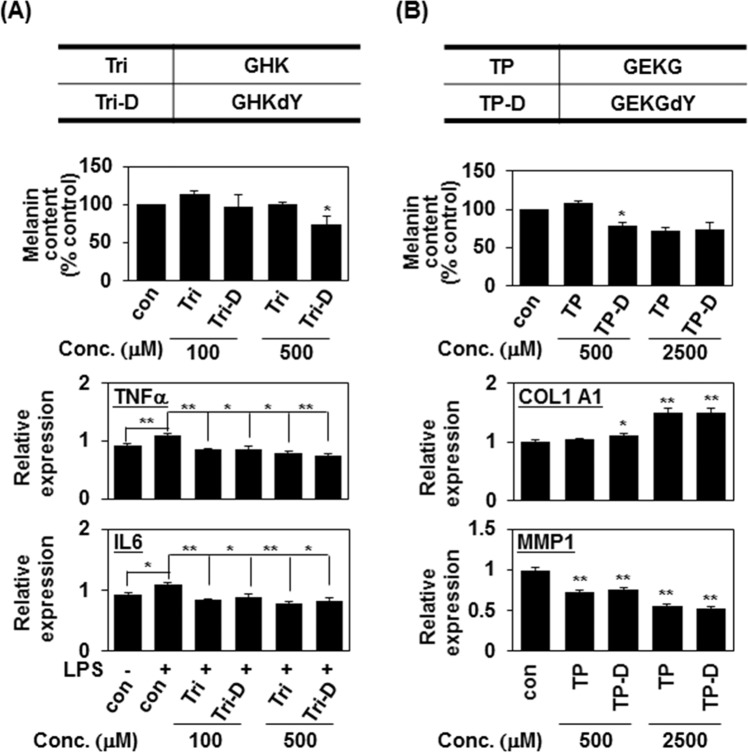
D-tyrosine confers an additional anti-melanogenic effect to short cosmetic peptides. (**A**) The anti-inflammatory tripeptide (GHK, Tri) was synthesized with an additional D-Tyr at the C-terminus (Tri-D). MNT-1 cells were treated with 100 μM and 500 μM of the indicated peptide for 24 h and the melanin content was analyzed. The mean percentages (n = 3) ± SD are shown; *P < 0.05 (top panel). Raw cells were treated with LPS (0.5 μg/ml) and the indicated concentration of peptides for 24 h, and the expression levels of the indicated mRNAs were analyzed by quantitative real-time PCR (qPCR). Data are shown as mean ± SD; *P < 0.05 and **P < 0.01 (bottom panel). (**B**) The anti-aging peptide, tetrapeptide-21 (GEKG, TP), was synthesized with an additional D-Tyr at the C-terminus (TP-D). MNT-1 cells were incubated with the indicated concentration of peptides for 24 h. The melanin content was measured by absorbance at 405 nm and is given as the mean of three independent experiments ± SD; *P < 0.05 (top panel). Primary human fibroblasts were treated with the indicated concentration of peptides for 24 h, and the expression levels of the indicated mRNAs were analyzed by quantitative real-time PCR (qPCR). Data are shown as mean ± SD; *P < 0.05 and **P < 0.01 (bottom panel).

Our results indicate that the terminal addition of D-tyrosine can give a peptide an additional anti-melanogenic effect. Since the addition of a single amino acid may interrupt the original function of a peptide, we investigated whether the addition of D-tyrosine had any effect on the inherent functions of several cosmetic peptides. As expected, 500 μM of the anti-inflammatory tripeptide, GHK (Tri), with a terminally added D-tyrosine also decreased the melanin content of both MNT-1 cells and the levels of pro-inflammatory cytokines (IL-6 and TNF-α) in RAW 264.7 cells (Fig. 5A), confirming that D-tyrosine can provide cosmetic peptides with an additional anti-melanogenic effect.

The tetrapeptide, GEKG (TP) known as an anti-aging peptide increased the productions of Type I collagen, hyaluronic acid synthase 1, and fibronectin to enhance skin elasticity^22^ and decrease facial wrinkles^17^. Consistent with a previous report^22^, 2,500 μM of GEKG peptide increased the mRNA expression of COL1A1 (collagen type 1 alpha 1) and reduced expression of MMP1 (matrix metalloproteinase 1) in primary human fibroblasts (Fig. 5B, bottom), confirming the anti-aging effect of the peptide. Interestingly, the corresponding D-tyrosine-containing peptides, TP-D, had anti-aging effects (Fig. 5B, bottom) and the anti-melanogenic effect (Fig. 5B, top), suggesting that D-tyrosine gives short commercial cosmetic peptides an additional anti-melanogenic effect.

### D-tyrosine confers an anti-melanogenic effect to short cosmetic peptides of less than five amino acid residues

**Figure 6:**
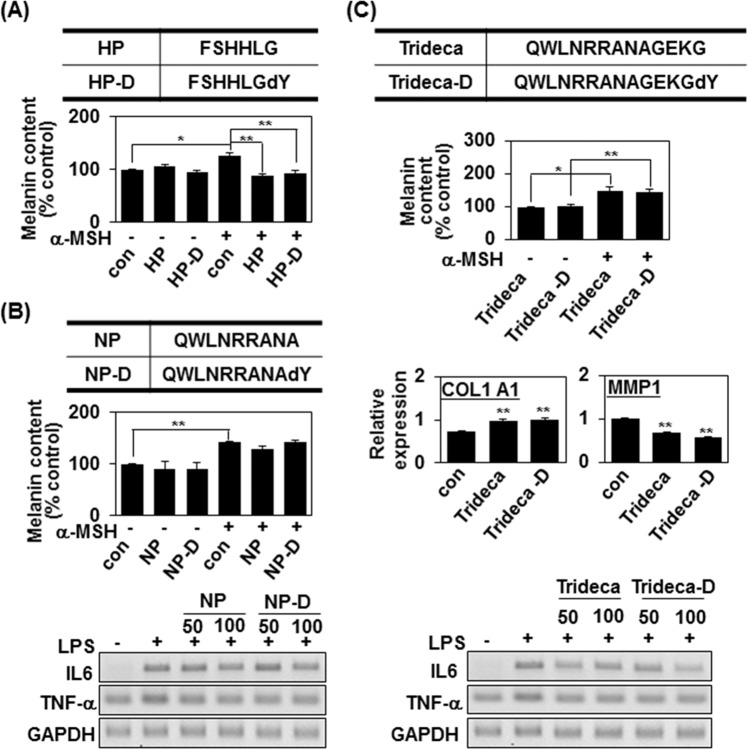
D-tyrosine confers an anti-melanogenic effect to short cosmetic peptides of less than five amino acid residues. (**A**) The anti-melanogenic hexapeptide (FSHHLG, HP) was synthesized with an additional D-Tyr at the C-terminus (HP-D). Anti-melanogenic activity of the peptide was analyzed as described in Fig. 5. The melanin content is given as the mean of three independent experiments ± SD; *P < 0.05 and **P < 0.01. (**B**) An anti-inflammatory nonapeptide (QWLNRRANA, NP) was synthesized with an additional D-Tyr at the C-terminus (NP-D). Both anti-inflammatory and anti-melanogenic activities of the peptide were analyzed as described in Fig. 5A. The mean percentages (n = 3) ± SD are shown; **P < 0.01. (**C**) Trideca peptide (QWLNRRANAGEKG) was synthesized with an additional D-Tyr at the C-terminus (Trideca-D). Anti-aging, anti-inflammatory and anti-melanogenic activities of the peptide were analyzed as described in Fig. 5B. The mean percentages (n = 3) ± SD are shown; *P < 0.05 and **P < 0.01.

Our results indicate that the terminal addition of D-tyrosine gave an anti-melanogenic effect to the tested cosmetic peptides, which were three to five peptides in length. However, some longer peptides are important for commercial use in cosmetics. Therefore, we further investigated whether D-tyrosine could offer an anti-melanogenic property when terminally added to longer peptides (Fig. 6). The hexapeptide, FSHHLG (HP), is known to attenuate melanin synthesis^23^. However, under our experimental conditions, the addition of D-tyrosine to HP at the C-terminus (HP-D) showed no additional effect on melanin synthesis in MNT-1 cells (Fig. 6A). The nonapeptide, QWLNRRANA (NP) is known as an anti-inflammatory peptide^24^. The generated peptide (NP-D) containing D-tyrosine at the C-terminus did not reduce the melanin content of MNT-1 cells under basal or α-MSH-stimulated conditions (Fig. 6B, top), although both NP and NP-D showed anti-inflammatory effects, as assessed by their ability to suppress LPS-induced IL-6 and TNF-α production (Fig. 6B, bottom). We also synthesized the synthetic Trideca-D peptide, which is composed of an anti-inflammatory peptide (NP) and an anti-aging peptide (TP), together with D-tyrosine at the C-terminus. However, 100 μM of Trideca-D peptide possessed both anti-inflammatory and anti-aging effects, but failed to reduce the melanin content of MNT-1 cells (Fig. 6C). Therefore, the anti-melanogenic effect of terminal D-tyrosine appears to apply to short peptides of probably less than five amino acid residues. Together, our data suggest that D-tyrosine at the C-terminus can confer an additional anti-melanogenic effect to a short cosmetic peptide without affecting its intrinsic function.

## Discussion

D-tyrosine is known to suppresses melanogenesis by inhibiting the enzymatic activity of tyrosinase^18^, but the effect of D-tyrosine-containing peptides on melanin synthesis was previously unknown. Here, we provide the first evidence that peptides containing terminal D-tyrosine, particularly at the C-terminus, down-regulate melanogenesis. Pentapeptide-18 (YdAGFL) containing D-tyrosine inhibited melanogenesis in human melanoma cells, human melanocytes, and the epidermal basal layer of a 3D human skin model (Figs. 1–4), indicating that the anti-melanogenic effect found for D-tyrosine as a free amino acid^18^ is retained when this residue is contained within a short peptide.

Importantly, the addition of D-tyrosine endows other short commercial cosmetic peptides with anti-melanogenic effects without altering their intrinsic functions. An example is the GEKG peptide, an anti-aging peptide due to its ability to induce type I collagen expression and reducing MMP1 expression^22^. GEKG peptides containing D-tyrosine at the C-terminus increased the mRNA expression of COL1A1 and reduce the expression of MMP1 in primary human fibroblasts (Fig. 5B), suggesting that the addition of D-tyrosine did not appear to affect the anti-aging effect of the GEKG peptide. Moreover, GEKG peptides containing D-tyrosine decreased melanin synthesis in MNT-1 cells (Fig. 5B). Similarly, GHK peptide, a known anti-inflammatory peptide, with terminal D-Tyr decreased the melanin contents of MNT-1 cells and reduced their levels of the pro-inflammatory cytokines in RAW 264.7 cells (Fig. 5A). These findings could have significant meaning for cosmetic applications: GEKG and GHK peptides containing terminal D-tyrosine could be used as dual-function cosmetic peptides having anti-aging/whitening and anti-inflammation/whitening effects, respectively. These data suggest that any short functional cosmetic peptide could be given an anti-melanogenic effect by the terminal addition of D-tyrosine, easily generating a dual-function peptide.

A peptide with multiple functions together with a whitening effect would offer significant advantages for commercial application. For example, atopic dermatitis is characterized by the simultaneous appearance of various symptoms, including dry skin, pigmentation, and inflammation. At present, various compounds must be mixed together to efficiently overcome these symptoms. In the future, the use of a D-tyrosine-modified anti-inflammatory peptide could offer both anti-inflammatory and anti-pigmentation effects. Similarly, multiple skin alterations occur with age; dermal changes (e.g., damage to elastic and collagen fibers) give rise to thickened, tangled, degraded, and non-functional fibers^25^, and the presence and extent of mottled pigmentation increases^26^. An anti-aging peptide linked with D-tyrosine could help improve both collagen degradation and skin pigmentation. Given that whitening is the basis of all cosmetics, D-tyrosine-modified peptides could prove very valuable in the cosmeceutical industry. It should be noted, however, that the anti-melanogenic effect of terminally added D-tyrosine appears to be limited to small cosmetic peptides, since the addition of D-tyrosine to longer peptide failed to endow these peptides with any anti-melanogenic effect (Fig. 6). We have previously shown that D-tyrosine directly inhibits tyrosinase by competitively inhibiting tyrosinase activity^18^. We therefore believe that the D-tyrosine residue in the peptide suppresses tyrosinase activity in the same way. In contrast to D-tyrosine as a free amino acid, there are other side chains attached to amino acid residues near the D-tyrosine residue in the D-tyrosine-containing peptides, and these side chains may interfere with access of the D-tyrosine residue to the active site of tyrosinase. However, this steric hindrance by the side chains of the peptide of 3 to 5 in length does not appear to sufficient to inhibit their interaction (Figs. 1–4). This may be consistent with the fact that D-tyrosine residue in the terminal region of the peptide, which is possibly more flexible, inhibited melanin synthesis (Fig. 1).

Peptides are more commercially viable than amino acids. Whereas amino acids have a low level of skin permeability, various skin-permeable peptides have been developed and can easily overcome the skin barrier. In addition, peptides are stable. These benefits have given them commercial appeal, particularly as skin care products. For instance, palmitoyl oligopeptide and acetyl hexapeptide-3 are used for collagen stimulation, wound healing, “Botox-like” wrinkle smoothing, and whitening effects^27^. Pentapeptide-18 is a neurotransmitter-inhibiting peptide that decreases neuronal activity and catecholamine release^28^, giving it Botox-like effects in reducing fine lines and wrinkles, and improving firmness^20^. Therefore, in terms of commercial applications, D-tyrosine-modified peptides hold more promise than D-tyrosine as a free amino acid. In addition, because of its dual functional role, D-tyrosine-modified peptide has the advantage of overcoming difficulties in mixing two peptides with different functions, thereby providing significant economic benefits.

In summary, we herein show for the first time that the addition of D-tyrosine at the C-terminus endows cosmetic peptides with an additional anti-melanogenic effect. We show that D-tyrosine-modified cosmetic peptides gain this skin-whitening effect while maintaining their original function and a commercially acceptable skin permeability. Although further studies will be required to fully elucidate the mechanism underlying this peptide-mediated inhibition of melanin synthesis, our present findings could guide cosmeceutical companies in developing new dual-function skin-whitening agents and multifunctional cosmetic peptides for therapeutic use.

## Materials and Methods

### Antibodies and materials

The polyclonal antibodies against tyrosinase and Trp1 and the monoclonal antibody against β-actin were purchased from Santa Cruz Biotechnology (Santa Cruz, CA, USA). The polyclonal antibody against MITF was purchased from Proteintech (Chicago, IL, USA). L-DOPA, melanin, and α-MSH were purchased from Sigma (St. Louis, MO, USA). Vivamagic was purchased from Vivagen (Seongnam, Korea). L-tyrosine and D-tyrosine were purchased from Sigma and dissolved in water with heating. Peptide synthesis was carried out by Anygen Inc. (Gwangju, Korea)^18^.

### Cell culture and transfection

The MNT-1 human melanoma cell line was maintained in minimal essential medium supplemented with 20% FBS, 10% DMEM, 20 mM HEPES, and 50 μg/ml gentamicin. The RAW 264.7 was maintained in DMEM (WelGene, Daegu-si, Korea), supplemented with 10% FBS with gentamicin (50 μg/ml). Human dermal fibroblasts (NHDF) from Lonza (Basel, Switzerland) were grown in FGM-2 fibroblast growth medium (Lonza) supplemented with 10% heat-inactivated FBS, 2 mM glutamine, 100 units/ml penicillin, and 100 µg/ml streptomycin. Human primary epidermal melanocytes purchased from Lonza were maintained in melanocyte growth medium-4 (Lonza) supplemented with FBS, rh-insulin, GA-1000 (gentamicin sulfate amphotericin-B), calcium chloride, PMA, bovine pituitary extract, hydrocortisone, and rh-FGF B. Transient transfections were carried out using Vivamagic according to the provided instructions^18^.

### RNA extraction and reverse transcription polymerase chain reaction (RT-PCR)

Total RNA was collected and reverse transcribed, and the resulting cDNA was amplified using the following primers: tyrosinase, 5′-CGAGCCTGTGCCTCCTCTAA-3′ (forward) and 5′-CCAGGACTCACGGTCATCCA-3′ (reverse); MITF, 5′-GGAACAGCAACGAGCTAAGG-3′ (forward) and 5′-TGATGATCCGATTCACCAGA-3′ (reverse); GAPDH, 5′-CAAGGTCATCCATGACAACTTTG-3′ (forward) and 5′-GTCCACCACCCTGTTGCTGTAG-3′ (reverse). After an initial denaturation at 94 °C for 5 min, the samples were subjected to 30 cycles of denaturation at 94 °C for 30 s, annealing at 55 °C for 30 s, and extension at 72 °C for 60 s^18^.

### Quantitative real-time PCR

Quantitative real-time PCR was performed using a CFX96 Real-Time PCR Detection System (Bio-Rad, Hercules, CA), in a two-step procedure applied with a SensiFAST SYBR Hi-ROX Kit (BioLine, London, UK). Glyceraldehyde-3-phosphate dehydrogenase (GAPDH) was amplified as an internal standard. The primer sequences were as follows: GAPDH, 5′-CCTCAAGATCATCAGCAAT-3′ (forward) and 5′-CCATCCACAGTCTTCTGGGT-3′ (reverse); COL1A1, 5′-GTCACCCACCGACCAAGAAACC-3′ (forward) and 5′-AAGTCCAGGCTGTCCAGGGATG-3′ (reverse); MMP1, 5′-CAGAGATGAAGTCCGGTTTTTC-3′ (forward) and 5′-GGGGTATCCGTGTAGCACAT-3′ (reverse); IL6, 5′-CCGGAGAGGAGACTTCACAG-3′ (forward) and 5′-TCCACGATTTCCCAGAGAAC-3′ (reverse); TNFα, 5′-ACGGCATGGATCTCAAAGAC-3′ (forward) and 5′-AGATAGCAAATCGGCTGACG-3′ (reverse). All reactions were performed in a 96-well plate using the following cycling conditions: 40 cycles of 95 °C for 15 s, 60 °C for 30 s, and 72 °C for 1 min. Using the CT (delta-delta CT) method^29^, the value of each control sample was set at 1 and used to calculate the fold changes of target genes.

### Immunoblotting

Cells were lysed in RIPA buffer (50 mM Tris, pH 8.0, 150 mM NaCl, 1% Nonidet P-40, 10 mM NaF, and 2 mM Na_3_VO_4_) containing a protease inhibitor cocktail (1 μg/ml aprotinin, 1 μg/ml antipain, 5 μg/ml leupeptin, 1 μg/ml pepstatin A, and 20 μg/ml PMSF). Cell lysates were clarified by centrifugation at 13,000 rpm for 15 min at 4 °C, denatured with SDS-PAGE sample buffer, and analyzed by SDS-PAGE. Proteins were transferred to 0.45-μm nitrocellulose blotting membranes (Amersham Biosciences, Piscataway, NJ, USA) and probed with the appropriate antibodies. Signals were detected with an Odyssey CLx imager (Lincoln, Nebraska, USA) and analyzed using the Image Studio Lite software (LI-COR Biosciences, Lincoln, NE, USA)^18,30^.

### Quantification of melanin

Cells on 6-well culture dish were washed twice with PBS, detached with 0.05% trypsin/EDTA, and collected by centrifugation. Thereafter, equal number of cells were solubilized in 100 μl of 1 N NaOH-10% DMSO at 80 °C for 2 h, and the melanin content of the solution was determined by measuring the absorbance at 405 nm and comparing the results to a standard curve generated with synthetic melanin (Sigma). The results were analyzed in percentage terms^30^.

### Tyrosinase activity assays

Cells were lysed in 50 mM sodium phosphate buffer (pH 6.8) containing 1% Triton X-100, 1 μM PMSF, 1 μg/ml aprotinin, and 10 μg/ml leupeptin. The lysates were clarified by centrifugation at 13,000 g for 15 min at 4 °C and reacted with L-DOPA in 50 mM sodium phosphate buffer (pH 6.8) at 37 °C for 3 h. Tyrosinase activity was determined by measuring the absorbance at 470 nm. For DOPA staining, cells were plated to coverslips in 12-well plates, fixed with 4% paraformaldehyde for 15 min, washed with PBS, and incubated in sodium phosphate buffer containing 10 mM L-DOPA for 3 h at 37 °C. The cells were then washed with PBS, the coverslips were mounted on glass slides, and the slides were observed and photographed with bright field microscope^30^.

### Cell proliferation assay

Cell proliferation was measured using the MTT [3-(4,5-dimethythiazol-2-yl) 2,5-diphenyltetrazolium bromide; Amresco, Solon, OH, USA] assay. In brief, after MNT-1 cells (5,000 cells/well) were incubated for 48 h, medium containing 0.5 mg/ml MTT was added to each well, and the cells were incubated for additional 1 h. The medium was then removed and 100 μl of acidic isopropanol (90% isopropanol, 0.5% sodium dodecyl sulfate (SDS) and 25 mM NaCl) was added to each well. The mean concentration of absorbance at 570 nm in each sample set was measured using a 96-well microtiter plate reader (Dynatech, Chantilly, VA, USA)^31^.

### UVB irradiation

For narrow-band UVB irradiation, we used a Dermalight 80 MED tester (National Biological Corp., Beachwood, OH, USA) with a broadband fluorescent UVB lamp (Philips PL 9W/12; Philips, Eindhoven, the Netherlands). The lamp emitted a wavelength spectrum from 305 to 315 nm, with a peak at 311 nm. During UVB irradiation, the lids of the dishes were removed and the culture medium was replaced with PBS to avoid the risk that UV exposure could trigger the formation of medium-derived toxic photoproducts. After irradiation (100 mJ/cm^2^ UVB), the PBS was replaced with medium^18,30^.

### *In vitro* 3D artificial skin model

MelanoDerm (MEL-300-B) (MatTek Corporation, Ashland, MA, USA) and the maintenance medium for EPI-100-NMM-113 were purchased from MatTek Corporation (Ashland, MA, USA). To measure melanin content, MelanoDerm tissue was incubated in 12 well plates containing the pre-warmed maintenance medium according to recommended protocols. The medium was changed every other day for 14 days^18^.

### Histological imaging

The MelanoDerm tissues were isolated from the inserts with the help of a fresh scalpel blade at specified time points and immediately fixed in 4% formalin and embedded in paraffin. Each paraffin block was sequentially sectioned at 4 μm, and the sections were mounted on slides. For measurement of melanin, the sections were stained using H&E and Fontana Masson (Abcam, Cambridge, UK) staining. A minimum of 20 fields per section was assessed using a color image analyzer (Leica DM1000 LED; Leica Microsystems, Wetzlar, Germany). Briefly, the images were captured, assess of melanin was counted by melanocyte per field of section X40 magnifications^18^.

### Statistical analysis

Data are presented as the mean obtained from three independent experiments. Statistical analyses were performed using an unpaired Student’s t-test or using the 1-way ANOVA followed by Bonferroni *post hoc* test. A P-value < 0.05 was considered statistically significant.

## Supplementary information

Supplementary Information.

## Data Availability

All relevant data are within the paper.
