# Supplementary material for: D-tyrosine adds an anti-melanogenic effect to cosmetic peptides
Source: Sci Rep. 2020 Jan 14;10:262. doi: 10.1038/s41598-019-57159-3 (PMC6959337; doi:10.1038/s41598-019-57159-3)
Supplement: Supplementary file 1 — Supplementary Information. [file 41598_2019_57159_MOESM1_ESM.pdf]

Supplementary information

D-tyrosine adds an anti-melanogenic effect to cosmetic peptides

Jisu Park[1], Hyejung Jung[2], Bohee Jang[1], Hyun-Kuk Song[1], Inn-Oc Han[3], and Eok-Soo Oh[1,2@]

Raw Data Figures

Figure S1

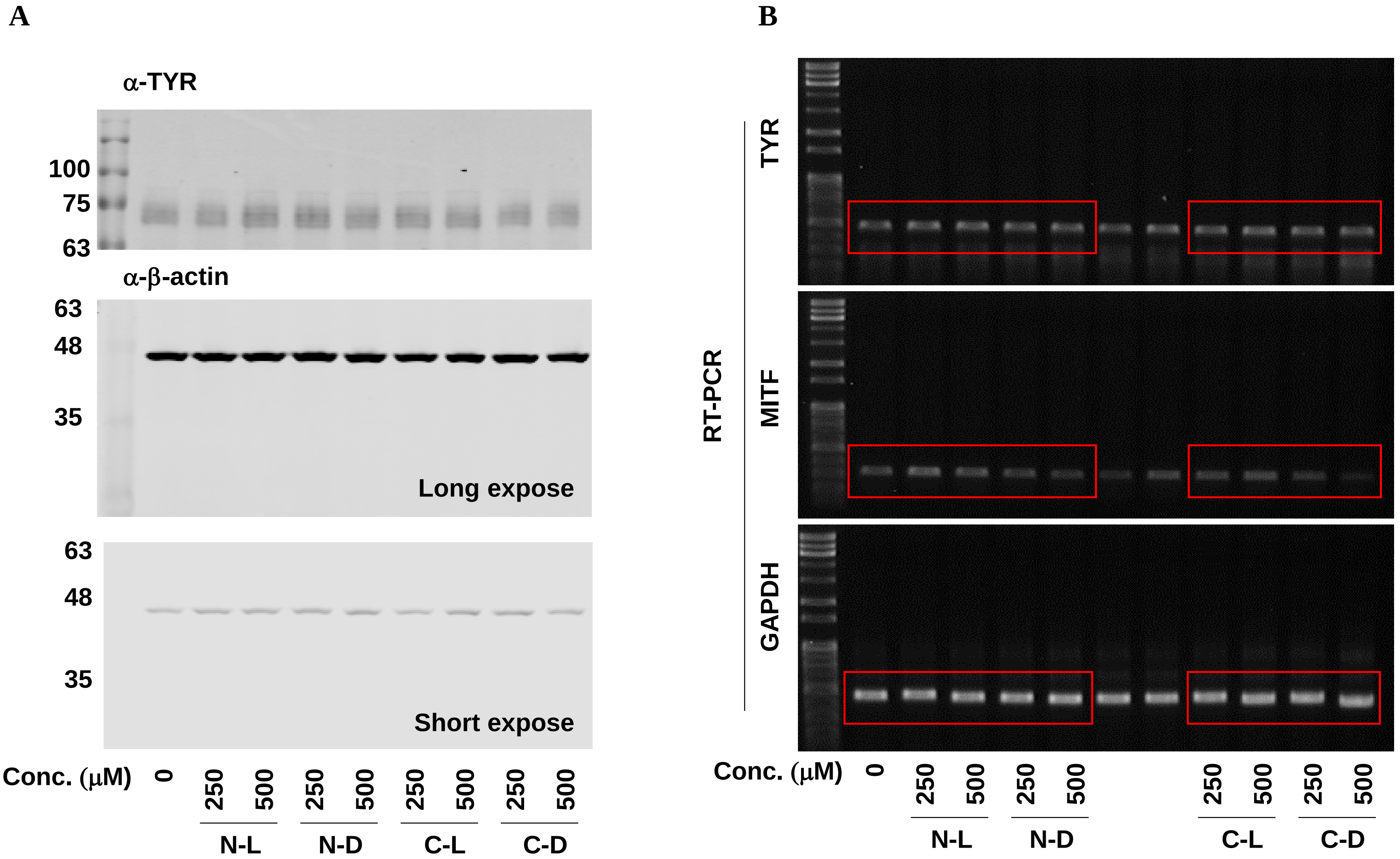

Supplementary Figure 1 : Full-length blot and gels presented in Figure 1.
Raw data of (A) Full length western-blots of α-TYR and α-β-actin. (B) Full length of agarose gel used for analysis of PCR products. (shown as cropped images in Figure 1 C).

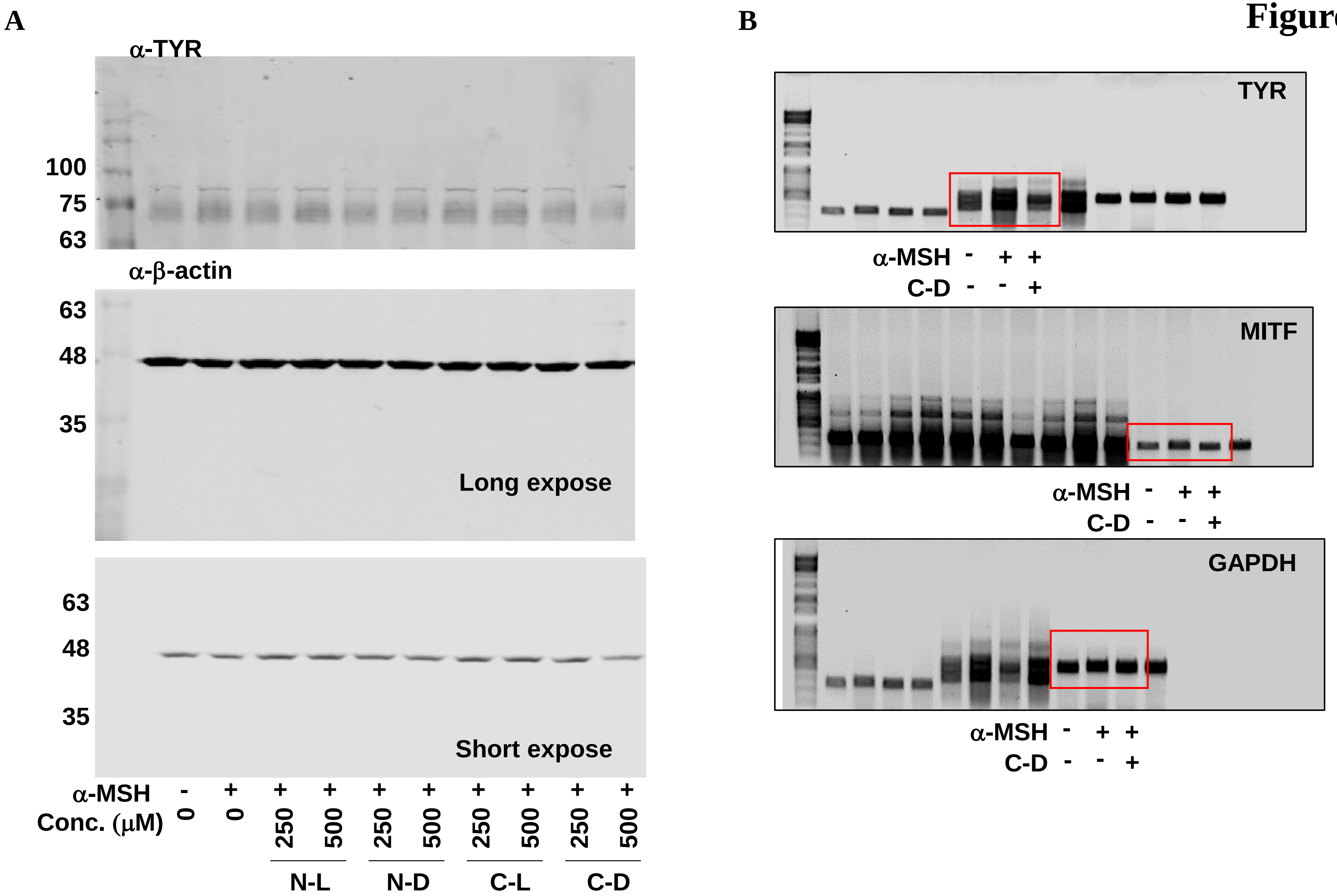

Supplementary Figure 2 : Full-length blot and gels presented in Figure 2A and Figure 2C. Raw data of (A) Full length western-blots of α-TYR and α-β-actin. (shown as cropped images in Figure 2 A).(B) Full length of agarose gel used for analysis of PCR products. (shown as cropped images in Figure 2 C).

Figure S3

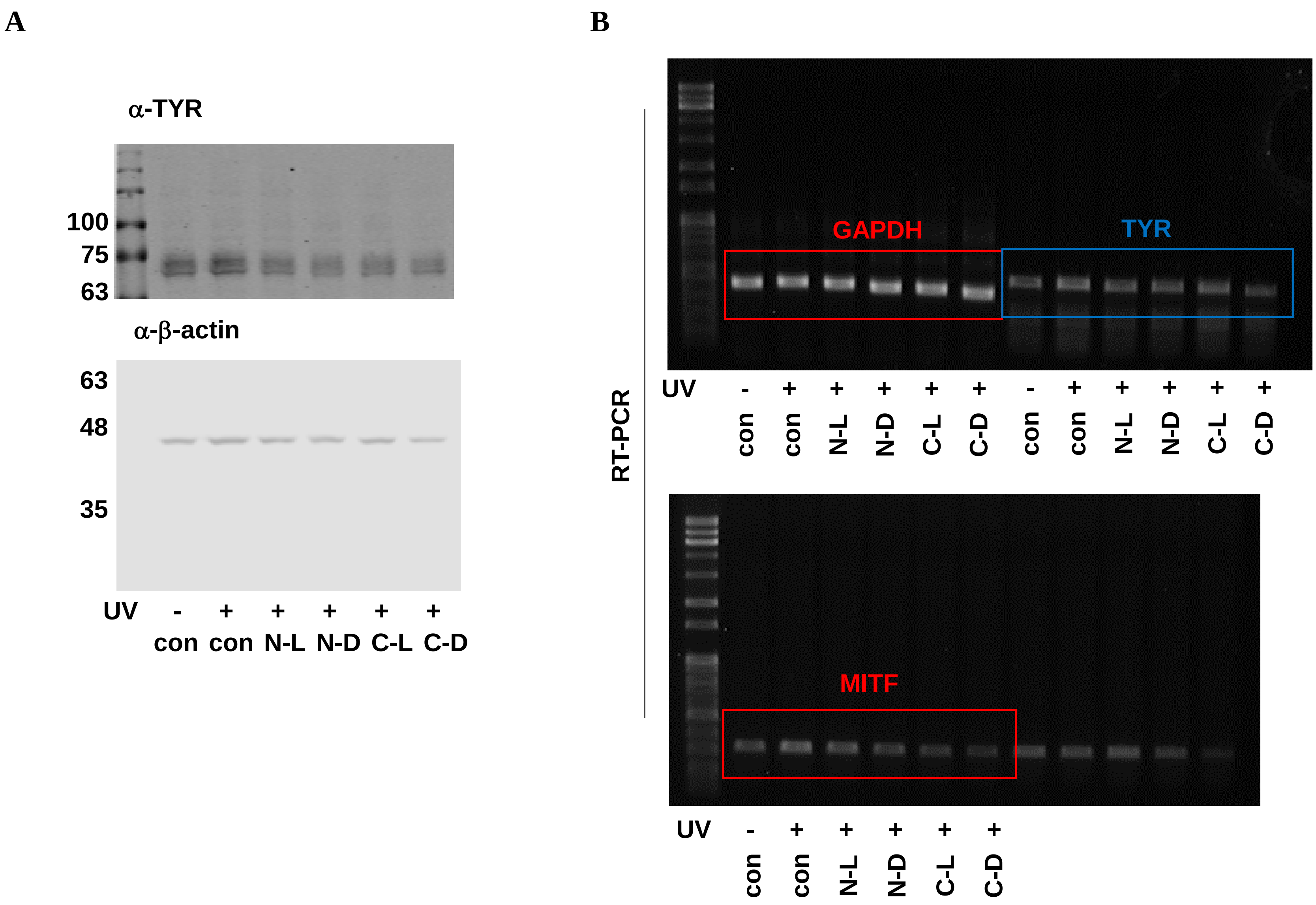

Supplementary Figure 3 : Full-length blot and gels presented in Figure 2B.
Raw data of (A) Full length western-blots of α-TYR and α-β-actin. (B) Full length of agarose gel used for analysis of PCR products. (shown as cropped images in Figure 2 B).

Figure S4

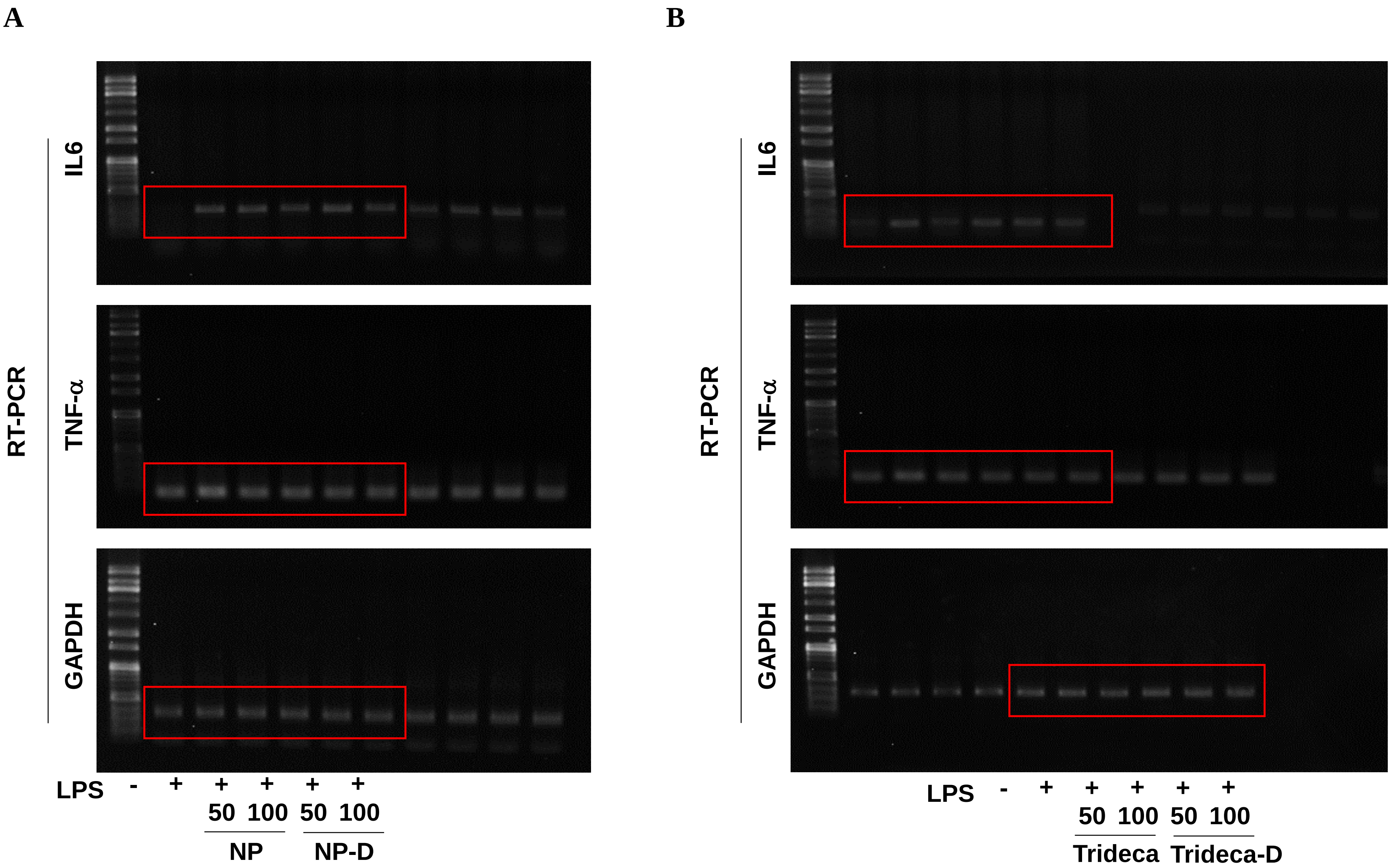

Supplementary Figure 4 : Full-length gels presented in Figure 6.

Raw data of (A, B) Full length of agarose gel used for analysis of PCR products. (shown as cropped images in Figure 6 B, C).
